# Community prescribing trends and prevalence in the last year of life, for people who die from cancer

**DOI:** 10.1186/s12904-022-00996-3

**Published:** 2022-07-08

**Authors:** Sarah E. E. Mills, Deans Buchanan, Peter T. Donnan, Blair H. Smith

**Affiliations:** 1 University of St Andrews, School of Medicine, North Haugh, St Andrews, KY16 9TF UK; 2 NHS Tayside, South Block, Level 7, Ninewells Hospital, DD2 4BF Dundee, Scotland; 3 Population Health and Genomics Division, University of Dundee Medical School Mackenzie Building, Ninewells Hospital and Medical School, Kirsty Semple Way, Dundee, DD2 4BF Scotland

**Keywords:** Prescribing, Palliative care, Cancer, Just in case medication, Opioids

## Abstract

**Background:**

People who die from cancer (‘cancer decedents’) may latterly experience unpleasant and distressing symptoms. Prescribing medication for pain and symptom control is essential for good-quality palliative care; however, such provision is variable, difficult to quantify and poorly characterised in current literature. This study aims to characterise trends in prescribing analgesia, non-analgesic palliative care medication and non-palliative medications, to cancer decedents, in their last year of life, and to assess any associations with demographic or clinical factors.

**Methods:**

This descriptive study, analysed all 181,247 prescriptions issued to a study population of 2443 cancer decedents in Tayside, Scotland (2013–2015), in the last year of life, linking prescribing data to demographic, and cancer registry datasets using the unique patient-identifying Community Health Index (CHI) number. Anonymised **l**inked data were analysed in Safe Haven using chi-squared test for trend, binary logistic regression and Poisson regression in SPSSv25.

**Results:**

In their last year of life, three in four cancer decedents were prescribed strong opioids. Two-thirds of those prescribed opioids were also prescribed laxatives and/or anti-emetics. Only four in ten cancer decedents were prescribed all medications in the ‘Just in Case’ medication categories and only one in ten was prescribed breakthrough analgesia in the last year of life. The number of prescriptions for analgesia and palliative care drugs increased in the last 12 weeks of life. The number of prescriptions for non-palliative care medications, including anti-hypertensives, statins and bone protection, decreased over the last year, but was still substantial. Cancer decedents who were female, younger, or had lung cancer were more likely to be prescribed strong opioids; however, male cancer decedents had higher odds of being prescribed breakthrough analgesia. Cancer decedents who had late diagnoses had lower odds of being prescribed strong opioids.

**Conclusions:**

A substantial proportion of cancer decedents were not prescribed strong opioids, breakthrough medication, or medication to alleviate common palliative care symptoms (including ‘Just in Case’ medication). Many patients continued to be prescribed non-palliative care medications in their last days and weeks of life. Age, gender, cancer type and timing of diagnosis affected patients’ odds of being prescribed analgesic and non-analgesic palliative care medication.

**Supplementary Information:**

The online version contains supplementary material available at 10.1186/s12904-022-00996-3.

## Background

Cancer already accounts for nearly a third of all deaths in the UK [1]. With cancer incidence increase outpacing improvements in survivability, the number of people dying from cancer each year is rising [2–4]. Over the course of their last year of life, people who go on to die from cancer develop an increasing level of disease activity, which can result in worsening pain and other distressing symptom [5].

In the UK palliative care is predominantly provided by community-based primary palliative care services [6, 7]. In Scotland, patients only spend an average of 21 days in hospital in their last six months of life; community prescribing data can therefore be expected to capture the majority of prescriptions issued to patients in their palliative phase [8]. A recent national survey identified effective symptom control through pharmacological management as one of the biggest challenges in delivering effective community palliative care [9]. As patients’ disease burden changes over the course of living with, and dying from, cancer, their medication requirements will also change, both with regards to starting new medication for new symptoms, and stopping existing medication which may no longer be of benefit to them. Pain is the most commonly-reported symptom in patients who go on to die from cancer, and is a significant catalyst for unplanned escalations in care, particularly towards the end of life [10–20]. Most important priorities for people with life-limiting illness, such as advanced cancer, are centred around being comfortable and free from pain and maximising quality of life [6, 21–23].

After pain, breathlessness [4, 10–15, 17, 19, 24, 25] and gastrointestinal symptoms [4, 10–15, 18, 19, 25] are the most commonly-reported symptoms associated with advanced or terminal cancer. While most people in the UK would prefer to die at home, the realities of a home death often involve worse pain and symptom control than many people find tolerable [22, 26, 27]. Though the majority of palliative care is provided at home, death at home is only achieved for a minority of people [8, 23]. Poor pain and symptom control is a significant factor in prompting admissions at the end of life; a large national survey found that only 19% of people who died at home had their pain adequately controlled, compared to 39% of people who died in hospital and 63% who died in a hospice [6]. Improving pain control through provision of analgesia in the community could address the disparity between pain control in hospitals and hospices compared to home. Having run out of prescribed pain medication was a significant factor contributing to avoidable escalations in care [10], with inadequate access to prescribed medication given as the commonest reason for acute hospital attendances among people with advanced cancer [28, 29]. Acute symptom management kits, containing anticipatory medication for predictable symptoms and side-effects patients may experience, can provide an effective way to manage palliative symptoms during the terminal phase of illness; they have been shown to reduce end-of-life hospital admissions, and prolonging time at home [30]. In the UK, such acute symptom management kids are provided in the form of Just in Case (JIC) Boxes [31].

There are robust national and international guidelines recommending which medications should be initiated to manage existing and future palliative care symptoms [32, 33].

The British National Formulary (BNF) contains UK-based guidelines on prescribing in palliative care [32], including the advice to discontinue any non-essential medication and to minimise the total number of drugs given. For pain, the WHO pain ladder’s stepwise progression from non-opioid, through weak opioid to strong opioid medication, has long been conventional for analgesics [34]. However, there is some suggestion that using low-dose strong opioids is preferable to using weak opioids in treating cancer pain [32, 33, 35, 36], and that pain due to cancer is often under-treated [37, 38]. Constipation, followed by nausea and vomiting, are the commonest side-effects of opioid medication reported by cancer decedents [37]. European Association for Palliative Care (EAPC) guidelines on Cancer Pain recommend that, in patients with incurable cancer, co-prescription of medication to prophylactically treat opioid side-effects should occur alongside opioid initiation, and that all patients with pain exacerbations should be treated with ‘additional doses of immediate-release oral opioids’ (commonly referred to as *‘breakthrough medication’*) [33]. For symptoms other than pain, BNF Palliative Care guidelines recommend the following treatment for common symptoms experienced by patients with advanced cancer: antimuscarinics for respiratory secretions and bowel colic; laxatives for constipation; morphine or diazepam for dyspnoea; and anti-emetics for nausea and vomiting [32].

Despite robust guidelines for prescribing for people with advanced cancer and palliative care needs, there are few real-world assessments of how often such medications are actually prescribed, or characterising the impact of demographic and clinical factors on prescribing.

This study aims to characterise the nature of prescription medication provided to cancer decedents, over their last year of life, and to identify how prescribing practices changed during that year and which factors were associated with prescribing practices.

## Methods

This descriptive study, analysed all 181,247 prescriptions issued to a study population of 2443 cancer decedents in Tayside, Scotland (2013–2015) in the last year of life, linking prescribing data to demographic, and cancer registry datasets using the unique patient-identifying Community Health Index (CHI) number. Anonymised **l**inked data were analysed in Safe Haven using chi-squared test for trend, binary logistic regression and Poisson regression in SPSSv25. The study population was identified posthumously using General Register Office death registration data and included all those whose recorded cause of death was cancer in position 1 of the death certificate. Unique patient-identifiable Community Health Index (CHI) numbers, which are attached to every patient registered with the National Health Service (NHS) in Scotland, were used to link demographic, cancer registry and clinical datasets with the national Community Prescribing Dataset (CPD). The CPD contains electronic records of all prescriptions issued in the community to each patient in Scotland; it includes all medication dispensed in the community, including medication initiated by secondary care (including oncology and palliative care specialists) on an outpatient basis, but does not include prescriptions issued in hospitals to inpatients. Prescribing information for all community prescriptions issued to each study population member in their last year of life was obtained from the CPD. Data were cleaned (addressing inconsistencies in formatting, labelline, duplication or corruption in source data), anonymised, stored and analysed in the Safe Haven platform in the Health Informatics Centre (HIC) at the University of Dundee. Individual medications were grouped into drug categories according to their BNF classifications (see Supplemental Materials Box S1 for classifications). Analysis deployed chi-squared testing and test for trend, multivariate and univariate logistic regression, and Poisson regression, and was conducted using SPSS v25.

For the purposes of this analysis, medication given in the last year of life has been divided into three broad categories: analgesia, non-analgesia palliative care medication, and non-palliative medication. Analgesia and non-analgesia palliative care medication will be collectively referred to as ‘palliative care medication’.

Logistic regression assessed demographic, cancer type and temporal factors associated with likelihood of being prescribed a particular class of drug, vs. not being prescribed that class of drug, in the last year of life. Factors included in logistic regression were: age, sex, cancer type, rurality (assessed using Scottish Government Urban Rural (SEUR) Classification data), deprivation (assessed using Scottish Index of Multiple Deprivation (SIMD) data) and time between diagnosis and death.

The sample size calculation shows that with a population of 2443 cancer decedents this study has 90% power in a logistic regression model to be able to detect odds ratios from 1.15 or above at the 5% significance level with a multiple correlation coefficient of 0.3 using the method of Hsieh [39].

Combination drugs which fit into more than one class, e.g. co-codamol, were counted in both categories (e.g. paracetamol and weak opioids) for the logistic regression models, which used binary outcomes of ‘prescribed’ vs. ‘not prescribed’ for each drug and drug class. For the Poisson models, as double-counting drugs in multiple categories would affect the numbers used in Poisson regression, drugs were allocated to their highest relevant class on the WHO pain ladder [34], e.g. ‘co-codamol’ would be allocated to ‘weak opioids’ and not to ‘paracetamol’. ‘Breakthrough medication’ was coded based on directions given (e.g. ‘as required’); drugs with variable prescription doses (e.g. take 1–2 tablets four times daily) were not included in ‘breakthrough medication’. Prescriptions were further analysed in broad categories of ‘Palliative Care Medication’ (which includes ‘Palliative analgesia’ and ‘Non-analgesic Palliative Care medication’) and ‘Non-Palliative Care Medication’.

## Results

A full descriptive analysis of the study population, in terms of age, gender, deprivation, rurality and cancer type, is available in the supplemental materials ‘S1: Description of Study Population Characteristics’.

### Number of prescriptions generated for cancer decedents in their last year of life

**Fig. 1:**
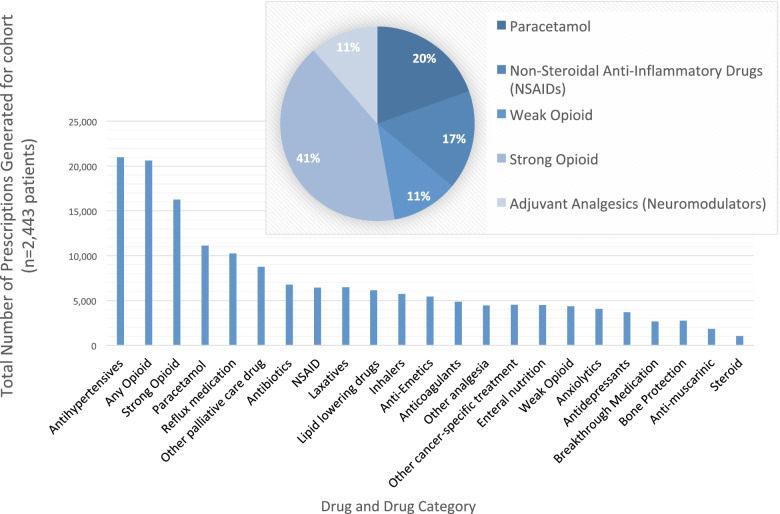
Prescriptions containing any form of drugs, by classification*. * In this chart, combination drugs were classed inclusively, e.g. co-codamol was counted in ‘weak opioids’ and ‘paracetamol’

There were 181,247 prescriptions generated for the 2443 cancer decedents in their last year of life. One quarter of these (*n* = 45,046 prescriptions) were for analgesia. Strong opioids were the most prescribed class of analgesia and accounted for nearly half of all analgesic prescriptions (Fig. 1). Anti-hypertensives, reflux medication, antibiotics, laxatives and lipid-lowering drugs were the most prescribed non-palliative care medication categories (Fig. 1).

### Number of cancer decedents prescribed each drug class in their last year of life

Among cancer decedents in their last year of life, two-thirds were prescribed a strong opioid (*n* = 1685 (69.0%)) and/or paracetamol (*n* = 1574 (64.4%)); half were prescribed NSAIDs (*n* = 1314 (53.8%)) and/or weak opioids (*n* = 1188 (48.6%)); and one third were prescribed another analgesic (*n* = 816 (33.4%)). Other palliative care prescriptions dispensed to cancer decedents in their last year of life included: three in five receiving laxatives (*n* = 1487(60.9%)) and/or anti-emetics (*n* = 1475 (60.4%)); half receiving anxiolytics (*n* = 1160 (47.5%)); two in five receiving antimuscarinics (*n* = 980 (40.1%)); and one quarter receiving cancer-specific treatment (*n* = 642, (26.3%)) and/or anti-depressants (*n* = 587 (24.0%)).

Antibiotics was the drug class prescribed to the most cancer decedents; three-quarters of cancer decedents (*n* = 1811 (74.1%)) received a prescription for antibiotics in their last year of life. Other non-analgesic or palliative care medications prescribed to cancer decedents in their last year of life included: nearly three-quarters receiving reflux medication (*n* = 1754 (71.8%)); two-thirds receiving anti-hypertensives (*n* = 1621 (66.4%)); half receiving steroids (*n* = 1380 (56.5%)); four in ten receiving lipid-lowering drugs (*n* = 976 (40.0%)); one quarter receiving anti-coagulants (*n* = 666 (27.3%)) and/or inhalers (*n* = 649 (26.7%)); and one in six receiving bone protection (*n* = 405 (16.6%)).

### Co-prescribing of medication

Eighty percent (*n* = 1972, 80.7%) of the cancer decedents in this study population were prescribed an opioid in their last year of life. Among cancer decedents prescribed strong opioids during their last year of life, 88.3% (*n* = 1741) were also prescribed paracetamol, 68.9% (*n* = 1358) were also prescribed anti-emetics, and 68.6% (*n* = 1352) were also prescribed laxatives. Only 10.9% (*n* = 214) of cancer decedents who were prescribed opioids were also co-prescribed breakthrough medication. There was no statistically significant difference between rates for co-prescription of breakthrough medications in cancer decedents who were prescribed weak opioids compared to those who were prescribed strong opioids. Over their last year of life, cancer decedents who were prescribed strong opioids were more likely to be co-prescribed anti-emetics (76.2% vs. 65.0%, *p* < 0.001) and laxatives (71.8% vs. 70.3%, *p* < 0.001) than those who were prescribed weak opioids.

### Prescribing trends over time during the last year of life

**Fig. 2:**
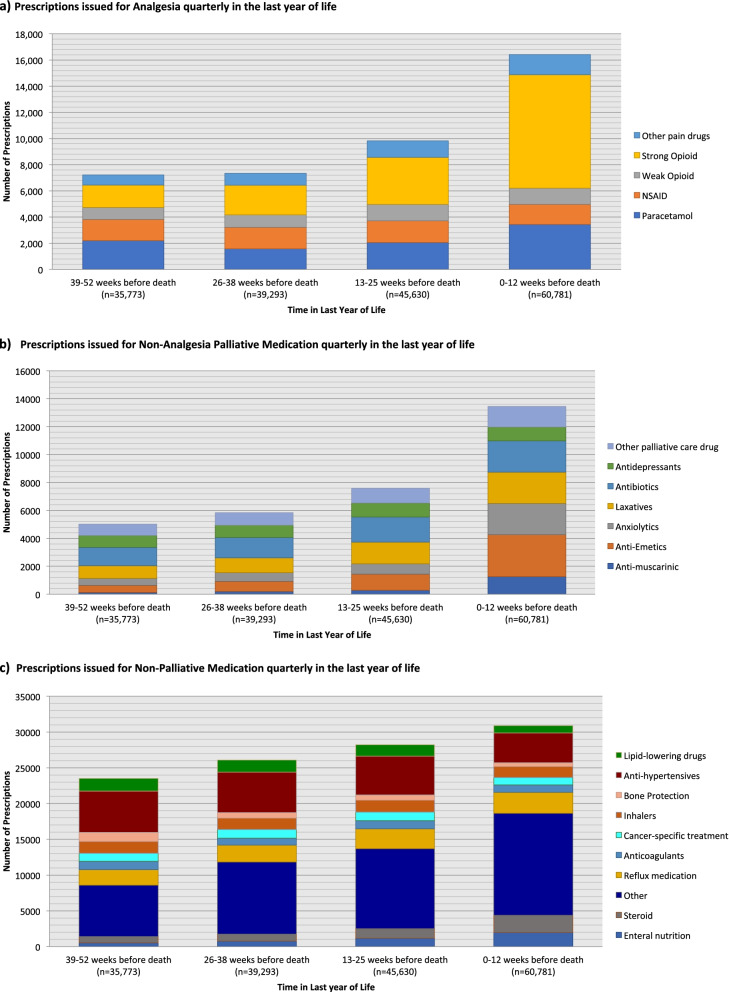
Prescriptions issued for Palliative and Non-Palliative Medications, quarterly, in the last year of life. **a** Prescriptions issued for Analgesia quarterly in the last year of life. **b** Prescriptions issued for Non-Analgesia Palliative Medication quarterly in the last year of life. **c** Prescriptions issued for Non-Palliative Medication quarterly in the last year of life

The number of prescriptions increased as patients neared the end of life (Fig. 2). This was true for both ‘palliative care’ (analgesia and non-analgesia categories) and ‘non-palliative care’ prescriptions, though the relative increase in number of prescriptions issued was much greater for palliative care prescriptions than for non-palliative care prescriptions, particularly in the last 12 weeks of life. Chi-squared values showed a significant (*p* < 0001) association between timing of prescription relative to death and total number of prescriptions issued, for all drugs and drug categories.

In cancer decedents’ last year of life, there was a 67% increase in number of prescriptions issued in the last quarter compared with the first quarter (Table 1). Over the last year of life, there was a five-fold increase in the number of strong opioid prescriptions, and a nearly two-fold increase in the number of paracetamol and other palliative care drug prescriptions. There was also a ten-fold increase in the number of anti-muscarinic prescriptions, a six-fold increase in the number of anti-emetic prescriptions, and a 4.5-fold increase in the number of anxiolytic prescriptions. There was a 20% increase in the number of antidepressant and antibiotic prescriptions, a 38% increase in the number of weak opioid prescriptions, and a small decrease in the number of prescriptions for NSAIDs.

**Table 1:** Number of prescriptions issued per quarter, to all 2443 cancer decedents, in their last year of life^a^

	Drug class	0–12 weeks before death (***n*** = 60,781)	13–25 weeks before death (***n*** = 45,630)	26–38 weeks before death (***n*** = 39,293)	39–52 weeks before death (***n*** = 35,773)	Relative increase^b^	OR (95%CI) Test for Trend (1st quarter vs. 4th quarter)
Analgesia and Non-Analgesic Palliative Care Medication.	Anti-muscarinic	1250	274	199	123	10.30	6.25 (5.26 to 7.69)***
	Anti-Emetics	3018	1169	731	515	6.00	3.70 (3.33 to 4.00) ***
	Strong Opioid	8668	3608	2254	1726	5.10	3.33 (3.23 to 3.57) ***
	Anxiolytics	2232	742	610	497	4.50	2.78 (2.50 to 3.03) ***
	Laxatives	2242	1554	1075	903	2.50	1.51 (1.41 to 1.64) ***
	Other pain drugs	1536	1254	925	768	2.00	1.22 (1.11 to 1.33) ***
	Other palliative care drug	1469	1058	902	802	1.86	1.11 (1.02 to 1.22) *
	Antibiotics	2245	1791	1449	1330	1.69	1.02 (0.67 to 1.10)
	Paracetamol	3444	2059	1580	2205	1.56	0.94 (0.89 to 1.00) *
	Weak Opioid	1241	1246	955	909	1.38	0.82 (0.75 to 0.90) ***
	Antidepressants	998	1004	883	844	1.20	0.71 (0.65 to 0.78) ***
	NSAID	1524	1658	1636	1618	0.94	0.56 (0.52 to 0.60) ***
Non-Palliative Care Medication	Enteral nutrition	1972	1173	782	555	3.60	2.17 (2.00 to 2.38) ***
	Steroid	2429	1339	956	847	2.88	1.75 (1.64 to 1.92) ***
	Other	14,258	11,181	10,087	7205	1.53	0.88 (0.86 to 0.92) ***
	Reflux medication	2926	2765	2377	2154	1.37	0.81 (0.77 to 0.86) ***
	Anticoagulants	1035	1157	980	1175	1.07	0.61 (0.56 to 0.66) ***
	Cancer-specific treatment	1024	1205	1184	1097	0.94	0.56 (0.51 to 0.61) ***
	Inhalers	1534	1648	1598	1662	0.94	0.55 (0.51 to 0.59) ***
	Bone Protection	557	735	763	1272	0.79	0.47 (0.42 to 0.52) ***
	Anti-hypertensives	4135	5407	5654	5790	0.71	0.39 (0.37 to 0.41) ***
	Lipid-lowering drugs	1044	1603	1713	1776	0.59	0.34 (0.32 to 0.37) ***

^a^In the following figures, the numbers of prescriptions for each medication class are presented over time. Time is presented as ‘weeks before death’, with ‘zero’ representing the date of death. Increasing time, shown on the X Axis, reflects time further away from time of death

^b^Relative Increase reflects the comparison in number of prescriptions issued per drug class between the last 0–12 weeks of life, and 39–52 weeks before death

Statistical significance: *: *p* < 0.05 **: *p* < 0.01 ***: *p* < 0.001.

**Fig. 3:**
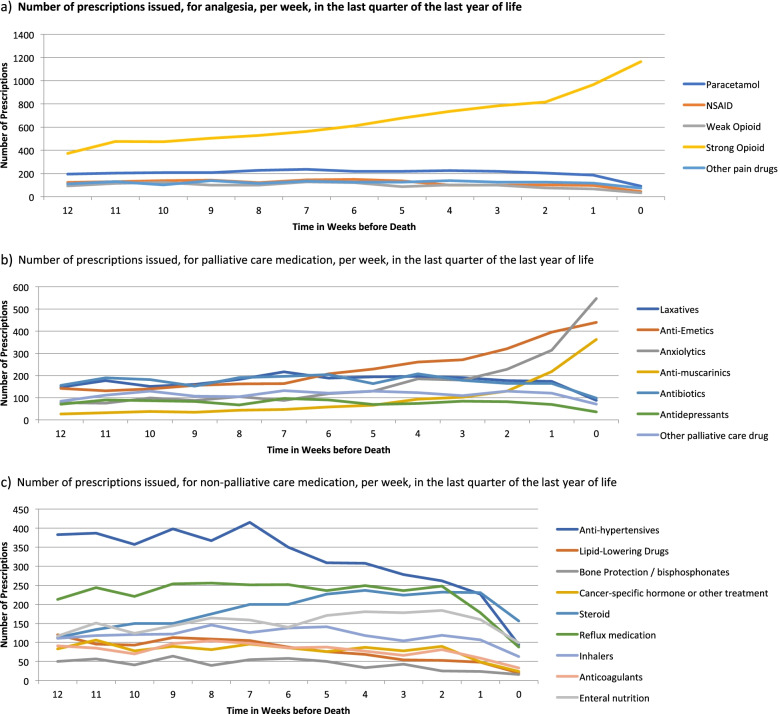
Number of prescriptions issued, per week, in the last quarter of the year of life. **a** Number of prescriptions issued, for analgesia, per week, in the last quarter of the year of life. **b** Number of prescriptions issued, for palliative care medication, per week, in the last quarter of the year of life. **c** Number of prescriptions issued, for non-palliative care medication, per week, in the last quarter of the year of life

In analgesic prescribing in the last year of life, the largest increase in prescribing was for strong opioids (Fig. 3). The number of prescriptions issued to cancer decedents for strong opioids increased five-fold between the first and last quarter of the year before death. There was an increase in the number of prescriptions for paracetamol and for other pain medications towards the end of life. Having stayed relatively static for most of the last year of life, there was a small decrease in NSAID prescribing rates in the last month of life (Fig. 3). There were significant changes in prescribing of non-analgesia medication over the last year of life, particularly medications which are used to treat palliative symptoms, or side-effects of opioids. Anti-muscarinic, anti-emetics and anxiolytics had the largest relative increase in prescribing close to the end of life. Prescribing of laxatives and steroids also increased close to the end of life (Fig. 3). There were decreases in prescribing of non-palliative care medications; however, many patients continued to be prescribed non-palliative care medications in their last weeks of life. Medications including anti-hypertensives and lipid-lowering drugs were prescribed less frequently, though did continue to be prescribed in the last months of life. Prescribing rates of some non-palliative care medications, e.g. antibiotics, increased during the last few months of life, then decreased in the last month of life (Fig. 3).

### Demographic factors associated with prescribing for Cancer decedents

On multivariate analysis, adjusting for the inter-relationship between demographic factors, cancer type and time between diagnosis and death, multiple statistically significant associations with prescribing were identified (Table 2). Men had lower odds of being prescribed paracetamol (AOR 0.89 (95%CI 0.86 to 0.93)) and any opioids (AOR 0.88 ((95%CI 0.86 to 0.91)) compared to women; however, men were more likely to be prescribed breakthrough analgesia^1^ (AOR 1.54 ((95%CI 1.40 to 1.69)) compared to women.

**Table 2:** Logistic regression assessed demographic, cancer type and temporal factors associated with likelihood of being prescribed a particular class of drug, vs. not being prescribed that class of drug, in the last year of life

*Factors used: age, sex, cancer type, rurality (SEUR), deprivation (SIMD) and time between diagnosis and death.* **Paracetamol**: Female sex; Lung and prostate cancer; Being diagnosed more than a year before death **NSAIDs**: Male sex; Older age; Having lung cancer; Being diagnosed close to the date of death **Any Opioid:** Female sex; Younger age; Having lung or prostate cancer; Living in accessible areas; Being diagnosed further from date of death **Weak Opioid:** Female sex, Younger age; Having lung or prostate cancer; Living in areas with more deprivation; Being diagnosed in the last 12 weeks of life **Strong Opioid:** Female sex; Younger age; Lung and prostate cancer; Being diagnosed before the last year of life **Breakthrough analgesia:** Male sex; Older age; Upper GI, Bowel, and Haematological malignancies **Other pain drugs:** Female sex; Younger age; Living in areas with less deprivation; Being diagnosed with cancer more than a year from date of death **Laxatives:** Male; Cancer type: upper GI, breast & ovarian, prostate cancer; Living in urban areas; Not having a late diagnosis **Anti-Emetics:** Female sex; Younger age; Upper GI, bowel and breast & ovarian cancers; Living in areas with less deprivation; Being diagnosed further from time of death. **Anxiolytics**: Female sex; Younger age; Having lung cancer; Living in urban areas; Living in areas with less deprivation; Being diagnosed closer to time of death **Antimuscarinics:** Younger age; Upper GI, and bowel malignancies; Living in accessible areas **Antibiotics:** Female; Being aged under 65 years old; Living in remote areas; Being diagnosed close to the date of death. **Antidepressants:** Female sex; Being aged under 65 years; Having haematological or breast and ovarian cancers; Living in urban areas; Being diagnosed with cancer close to the date of death **Other palliative care drug:** Younger age; Upper GI malignancy; Living in remote areas; Not having a late cancer diagnosis **Anti-hypertensives:** Older age; Having haematological malignancies; Living in accessible areas; Living in areas with less deprivation; Being diagnosed close to the end of life **Lipid-lowering drugs:** Male sex; Older age; Breast and ovarian cancer; Being diagnosed closer to the end of life **Bone Protection**: Female sex; Increased age; Breast and ovarian cancer, haematological malignancies; Living in urban areas; Being diagnosed closer to date of death **Cancer-specific treatment**: Male sex; Increased age; Having Upper GI, breast & ovarian, prostate, and/or haematological malignancies; Living in urban areas; Living in areas with less deprivation. **Steroids:** Younger age; Lung cancer; Living in accessible or remote areas; Living in less deprived areas; Not having a late diagnosis **Reflux medication:** Upper GI malignancy; Living in less deprived areas **Inhalers:** Older age; Lung cancer; Living in remote areas; Living in areas with more deprivation; Late diagnosis **Anticoagulants:** Male sex; Older age; Having breast and ovarian cancers; Living in areas with less deprivation **Enteral nutrition:** Men; Younger age; Upper GI and bowel malignancies (more likely); Breast and ovarian (less likely); Cancer decedents who live remotely; Being diagnosed further from date of death	

Older cancer decedents were significantly less likely to be prescribed opioids compared to younger ones; when compared to those aged under 65 years old, those aged 64–74 were 30 % less likely to be prescribed opioids (AOR 0.73 (0.70 to 0.76)), those 75–84 were fifty-percent less likely to be prescribed opioids (AOR 0.51 (0 .49 to 0.53)) and those aged over 85 were over 60 % less likely to be prescribed opioids (AOR 0.37 (0.35 to 0.39)). Cancer decedents who died from lung or prostate cancer were more likely to have been prescribed opioids than those with other cancers. Younger cancer decedents (aged under 65 years) were more likely to be prescribed palliative symptom control medication, including anti-emetics (AOR 2.13 ((95%CI 1.92 to 2.34)), anxiolytics (AOR 2.22 ((95%CI 1.96 to 2.44)), and antimuscarinics (AOR 2.17 ((95%CI 1.85 to 2.5)) and other palliative care medication (AOR 1.56 ((95%CI 1.41 to 1.72), than older cancer decedents (aged over 85 years).

Cancer decedents’ type of cancer was significantly associated with their odds of being prescribed every class of medication examined. In general, patients with lung cancer were more likely to receive medication than those with other cancer types. Notably, people with prostate cancer were more likely to receive either weak opioids (AOR1.24 ((95%CI 1.07 to 1.45)) or strong opioids (AOR1.09 ((95%CI 1.01 to 1.18)), people with haematological malignancies were most likely to be prescribed breakthrough medication (AOR1.60 ((95%CI 1.39 to 1.86)), people with Upper GI malignancies were most likely to receive anti-emetics (AOR1.83 ((95%CI 1.69 to 1.98)), and people with prostate (AOR3.13 ((95%CI 2.80 to 3.50)) and breast/ovarian cancers (AOR) 3.69 ((95%CI 3.26 to 4.18)) were more likely to receive cancer-specific medication, than people with lung cancer. Full multivariate analysis results available in the supplemental materials, Table S1.

Cancer decedents who were diagnosed close to the end of life were nearly three times less likely to receive community prescriptions for strong opioids than those diagnosed more than a year before death (AOR2.44 ((95%CI 2.32 to 2.56)).

## Discussion

While the proportion of this study population who received strong opioids was higher than has been found in previous studies [37, 38], a third were not prescribed strong opioids in their last year of life. This may reflect that patients achieved good pain control on non-opioid analgesia, that opioids were contraindicated, or that the patients declined opioid analgesia [40]; however, given the prevalence of pain in advanced cancer, it is also possible that these patients had unmet pain treatment needs and may have benefited from access to strong opioids in their end of life care [5, 30, 33]. The higher rate of strong opioid prescribing seen in this study may be due to previous studies have examined all patients with cancer, whereas this study selected patients who died from cancer and who were in their last year of life. Patients with advanced or terminal cancer would be expected to have a higher symptom burden than those for whom cancer is curable or is a chronic stable condition for many years.

In this study, the majority of patients on opioids were prescribed prophylactic treatment to manage side-effects of opioid use, including constipation and nausea; this rate of co-prescribing is higher than has been seen in other similar studies [32, 33, 37]. However, this rate of co-prescription still means one-third of patients on opioids did not receive prescription medication to manage common and predictable opioid-related side-effects. It is possible that these patients did not experience nausea, vomiting or constipation, that they preferred to use non-pharmacological methods for addressing these symptoms, or that they had contraindications to these co-prescriptions. However, given the ubiquity of such symptoms, it is also possible that these patients did experience side-effects of opioids, and might have benefited from anti-emetics and laxatives being available [5]. Further investigation of the causality behind lack of co-prescribing is needed to characterise this finding.

While there is no specific prescribing code to identify ‘Just in Case’ (JIC) medication provision, JIC boxes typically contain four medications: strong opioids, anti-emetics, anxiolytics and anti-muscarinics. While strong opioids, anti-emetics and anxiolytics are often prescribed independently of JIC boxes, anti-muscarinic medication is probably infrequently prescribed outside of JIC medications. Using anti-muscarinic prescriptions as a proxy for JIC prescribing, it suggests that only 40% of cancer decedents died with access to JIC medication in the community. Similarly, only a minority of patients who received prescriptions for strong opioids were prescribed breakthrough analgesia to manage pain escalations. While guidelines suggest that patients in their end-of-life phase should be prescribed all JIC medications, it is possible that some cancer decedents were prescribed ad hoc JIC medication without being provided the full complement of strong opioids, anti-emetics, anxiolytics and anti-muscarinics. However, in this study less than half of patients received anxiolytics and 60% received anti-emetics, which suggests that even partial JIC prescriptions would still be absent in at least 40% of cancer decedents. Improving rates of prescribing for JIC and breakthrough medication could yield substantial improvements in pain control and quality of life for patients dying from cancer. Developing specific data markers for JIC medication would give future research in this area a more complete picture of JIC prescribing.

While the reduction in the prescribing rates of non-palliative medication over cancer decedents’ last year of life is reflective of good practice, many patients in this study continued to receive medication, e.g. lipid-lowering drugs, which were unlikely to confer any clinical benefit in the context of their terminal illness, and which may therefore be considered unnecessary at best or harmful at worst. The increase seen in steroid prescribing towards the end of life likely reflects instances in which they were used in palliative or acute oncological event settings.

Demographic factors which influenced cancer decedents’ chances of having been prescribed opioids, breakthrough medication and JIC medication included age, gender, and timing of diagnosis relative to death. In this study, younger cancer decedents were more likely to be given prescriptions for JIC medication, and for other palliative care medication, than older cancer decedents. Despite women receiving more prescriptions per person for opioids, men were more likely to be given breakthrough analgesia. This may be due to men relying on breakthrough analgesia rather than regular analgesia; however palliative care guidelines recommend that any patient receiving regular opioids should also be co-prescribed breakthrough medication for escalations in pain [32]. Relative under-prescribing of breakthrough analgesia has been recognised in other research in this field [37]. Such age- and gender-based variation in prescribing is a significant potential area for prescribing inequality in cancer care, which bears further investigation.

Some trends in prescribing mirrored predictable cancer-specific symptoms or sequelae. For example, compared to patients with other cancer types, those with lung cancer were more likely to be prescribed steroids, those with Upper GI malignancies were more likely to be prescribed reflux medication; and those with bowel or Upper GI malignancies were more likely to be prescribed enteral nutrition.

Cancer decedents with a late diagnosis were substantially less likely to receive community prescriptions for strong opioids than those who did not have a late diagnosis; this may reflect a lack of time for appropriate anticipatory care planning and prescribing between diagnosis and death, or may be due to people with a late diagnosis also being more likely to receive this diagnosis during a hospital admission. Cancer decedents who are diagnosed and die within a single admission may receive strong opioid medication in hospital, which would not be apparent from community prescribing records. In newly-diagnosed patients with advanced disease, or other features suggesting a late diagnosis, in the community, it may be more appropriate to use strong opioids immediately from the time of diagnosis, rather than trialling weak opioids first [33, 35].

Optimising prescribing by initiating appropriate analgesia and palliative medications and discontinuing unnecessary and potentially harmful non-palliative medications should improve symptom control and improve quality of life for people dying from cancer.

## Strengths and limitations

Through using retrospective data, this analysis was able to include a comprehensive picture of medication prevalence and trends in community prescribing for a study population of people who died from cancer.

One limitation of this study is the identification of ‘breakthrough’ medication. There is no data coding that reflects whether medication are used for breakthrough pain or not, and while certain medication are more likely to be used as breakthrough medications, e.g. fast-acting strong opioids, there is no universally agreed definition for what medications constitute ‘breakthrough’ analgesia. For this analysis, ‘breakthrough medication’ was defined based on directions given, and drugs that contained the description ‘as required’ were used as breakthrough medication. However, this would exclude drugs for which there was a variable dose range (e.g. take 1–2 tablets up to four times daily) where that dose variation may have been used as ‘breakthrough’ analgesia. This may lead to under-reporting of the prevalence of breakthrough analgesia in this population. It should also be noted that this is a prescribing-level analysis and that there is no correlation possible with patients’ clinical condition and symptoms, their quality or life, or their wishes with regards to their care; the absence of prescribing of breakthrough medication, symptom control medication (e.g. laxatives and anti-emetics) and strong analgesia may be due to patients having achieved satisfactory symptom control. It may also be reflective of patients’ wishing to avoid sedation or medication at the end of life. In cases of prescribing that has the potential not to confer benefit, such as lipid-lowering drugs, this may be due to late diagnoses where there was not sufficient time to put in place palliative anticipatory care planning. In cases where patients continued to be prescribed medication with a curative intent, e.g. antibiotics or cancer-specific treatments, this may be reflective of the patient’s expressed wishes, or to improve the symptom burden of cancer or other illnesses for comfort in a dying patient. Interpreting patients’ prescribing records in conjunction with their clinical records could help further elucidate the reasons behind the observed prescribing trends.

While it’s completeness in capturing all prescriptions generated in the community makes it a robust description of community prescribing, it does not contain data related to hospital prescribing. It is therefore possible that patients received medications in hospital, including strong opioids and palliative care medication, which would not have been identified in this community prescribing analysis. For patients with cancer types associated with higher rates of hospital admission, including lung and pancreatic cancers, the absence of hospital-level prescribing data may lead to under-reporting of medications received [41]. However, in the Health Board area examined in this study, during the years in question, patients spent 89.8% of their last six months of life in the community, suggesting that community prescribing datasets would still capture the vast majority of prescribing that occurs in the last year of life [42]. Furthermore, in Scotland, rates of hospitalisation are largely independent of the demographic factors studied, which substantially reduces this potential source of bias. In Scotland, age and gender had no significant impact on number of days spent in hospital in the last year of life [42]. There were subtle variations to this, with women aged > 85 years and men aged < 55 years spending an average of 4–5 days more of their last six months of life at home in the community, when compared to other age and gender groups [42]. Rurality and deprivation showed no significant impact on amount of time spent in hospital in the last year of life [42].

One limitation to this study is that it related to a limited region within Scotland; however, the demographics of this observed population are approximately representative of Scotland as a whole, and the nationalised healthcare system available in this area is similar to that of the UK as a whole, and some other European countries. It is therefore likely that the findings of this paper are applicable and generalisable to healthcare delivery, community prescribing, and end-of-life care throughout the UK and in countries with similar demographics and healthcare systems. The large proportion of time that patients in Scotland spend in the community gives a high proportion of completeness to this descriptive study; however, may limit its generaliseability to populations where the majority of end of life care is delivered in inpatient, or non-community, settings. Similar studies in such settings would prove an interesting point of comparison as to the impact of hospitalisation and institutionalisation on prescribing in the last year of life.

## Conclusions

A substantial proportion of cancer decedents in this study population died without having been prescribed strong opioids, breakthrough medication, or medication to alleviate common palliative care symptoms (including JIC medication). Many patients continued to be prescribed potentially unnecessary non-palliative care medications in their last weeks of life, including up to their date of death. Age, gender, cancer type and timing of diagnosis affected patients’ odds of being prescribed many analgesic and non-analgesic palliative care medication. These factors should form the basis of future prescribing interventions aimed at improving adherence to good prescribing practice for patients dying from cancer.

## Supplementary Information

**Additional file 1.**

**Additional file 2.**

## Data Availability

The datasets generated and/or analysed during the current study are not publicly available due to using deidentified but individual level healthcare data which is accessible via Health Informatics Centre, Dundee but are available from the corresponding author on reasonable request.

## Abbreviations

A&E: Accident and Emergency department
AOR: Adjusted Odds Ratio
BNF: British National Formulary
CI: Confidence Interval
EAPC: European Association for Palliative Care
GRO: General Registry Office
HIC: Health Informatics Centre, University of Dundee
ICD10: International Classification of Diseases 10th revision, produced by the World Health Organisation (WHO). It assigns codes to particular diseases and conditions.
JIC: Just in Case (*refers to Just in Case medication or Just in Case boxes*)
NHS: National Health Service
NICE: National Institute for Health and Care Excellence
NRS: National Records of Scotland
NSAID: Non-Steroidal Anti-Inflammatory Drug
OR: Odds ratio
PIS: Prescribing Information System
SEUR: Scottish Executive Urban Rural Classification
SIMD: Scottish Index of Multiple Deprivation
WHO: World Health Organisation

## Footnotes

1 Breakthrough analgesia are defined as opioid analgesia where the prescription instructions state ‘as required’
