# Supplementary material for: Community prescribing trends and prevalence in the last year of life, for people who die from cancer
Source: BMC Palliat Care. 2022 Jul 8;21:120. doi: 10.1186/s12904-022-00996-3 (PMC9264643; doi:10.1186/s12904-022-00996-3)
Supplement: Supplementary file 1 — Additional file 1. [file 12904_2022_996_MOESM1_ESM.docx]

**Supplemental Materials**

**Box S1: Classification and Allocation of Drugs into ‘Drug Categories’**

| **Anti-emetics:** betahistine dihydrochloride, cyclizine, domperidone, granisetron hydrochloride, levomepromazine, metoclopramide hydrochloride, ondansetron, prochlorperazine.  **Anti-hypertensives:** amiloride hydrochloride, amiodarone hydrochloride, amlodipine, atenolol, bendroflumethiazide, betaxolol hydrochloride, bisoprolol fumarate, bumetanide, candesartan cilexetil, carvedilol, celiprolol hydrochloride, chlortalidone, co-amilofruse, co-amilozide, co-tenidone, co-zidocapt, diltiazem hydrochloride, doxazosin, enalapril maleate, eplerenone, felodipine, fosinopril, furosemide, hydralazine hydrochloride, indapamide, irbesartan, irbesartan and hydrochlorothiazide, isosorbide dinitrate, isosorbide mononitrate, lacidipine, lercanidipine, lisinopril, lisinopril and hydrochlorothiazide, losartan potassium, losartan with hydrochlorothiazide, metolazone, metoprolol tartrate, moxonidine, nebivolol, nicardipine hydrochloride, nifedipine, olmesartan medoxomil, perindopril, perindopril with indapamide, pravastatin, prazosin hydrochloride, propranolol hydrochloride, quinapril, ramipril, spironolactone, telmisartan, torasemide, trandolapril, triamterene with furosemide, valsartan, valsartan and hydrochlorothiazide, verapamil hydrochloride  **Anti-muscarinics:** aclidinium bromide, alverine citrate, amfebutamone, atropine sulfate, cyclopentolate hydrochloride, dicycloverine hydrochloride compound preparations, erdosteine, fesoterodine, flavoxate hydrochloride, glycopyrronium bromide, hyoscine butylbromide, hyoscine hydrobromide, hyoscine hydrobromide, procyclidine hydrochloride, propantheline bromide, propiverine hydrochloride  **Antibiotics:** amoxicillin, azithromycin, betamethasone with fusidic acid, betamethasone with neomycin, betamethasone with salicylic acid, cefalexin, cefuroxime, chloramphenicol, ciprofloxacin, clarithromycin, clindamycin, co-amoxiclav, co-fluampicil, co-trimoxazole, dapsone, demeclocycline hydrochloride, dexamethasone with framycetin and gramicidin, doxycycline, erythromycin, flucloxacillin, fluocinolone acetonide with neomycin, fusidic acid, gentamicin, gentamicin with hydrocortisone, hexetidine, hydrocortisone with fusidic acid, isoniazid, levofloxacin, lymecycline, metronidazole, moxifloxacin, neomycin sulfate, nitrofurantoin, norfloxacin, ofloxacin, oxytetracycline, phenoxymethylpenicillin, pivmecillinam, rifaximin, sulfadiazine, tetracycline, trimethoprim  **Anti-coagulants:** clopidogrel, dalteparin sodium, dipyridamole, dipyridamole with aspirin, enoxaparin, etamsylate, Indacaterol, indacaterol with glycopyrronium bromide, ipratropium bromide, rivaroxaban, warfarin sodium  **Anti-depressants:** citalopram, clomipramine hydrochloride, dosulepin hydrochloride, doxepin, escitalopram, fluoxetine, fluvoxamine maleate, imipramine hydrochloride, lofepramine, mirtazapine, moclobemide, paroxetine, sertraline, trimipramine, venlafaxine  **Anxiolytics:** buspirone hydrochloride, chlordiazepoxide, clobazam, clomethiazole, clonazepam, diazepam, loprazolam, lorazepam, lormetazepam, midazolam, midazolam hydrochloride, nitrazepam, temazepam, trazodone hydrochloride, zaleplon, zolpidem  **Bone protection:** alendronic acid, calcium acetate, calcium carbonate, calcium polystyrene sulphonate, calcium salts, calcium with vitamin d, denosumab, ibandronic acid, risedronate sodium  **Cancer-specific treatment:** alfuzosin hydrochloride, bicalutamide, cyproterone acetate, degarelix, dutasteride, dutasteride and tamsulosin hydrochloride, estradiol, estradiol with norethisterone, estriol, exemestane, finasteride, fluorouracil, hydroxycarbamide, imiquimod, lanreotide, letrozole, leuprorelin acetate, megestrol acetate, mercaptopurine, mirabegron, octreotide, octreotide acetate, oestrogens conjugated, oxybutynin hydrochloride, solifenacin, tamoxifen, tamsulosin hydrochloride, testosterone, testosterone undecanoate, tolterodine, triptorelin, trospium chloride  **Enteral nutrition:** enteral nutrition  **Inhalers:** fluticasone furoate and vilanterol, fluticasone propionate, formoterol fumarate, mometasone furoate, montelukast, nedocromil sodium, salbutamol, salbutamol with ipratropium, salmeterol, salmeterol with fluticasone propionate, terbutaline sulfate, theophylline, tiotropium, budesonide with formoterol fumarate  **Laxatives:** activated simethicone, anal irrigation systems, Bisacodyl, co-danthramer, co-danthrusate, docusate sodium, fenofibrate, ispaghula husk, ispaghula husk with mebeverine, ispaghula with senna, lactulose, macrogols, methylnaltrexone bromide, micro-enema sodium citrate, senna, sodium picosulfate, sodium picosulfate and magnesium citrate  **Non-Steroidal Anti-Inflammatory Drugs (NSAIDs):** aceclofenac, aspirin, benzydamine hydrochloride, celecoxib, coal tar with salicylic acid and sulphur, compound preparations with salicylic acid, diclofenac, diclofenac with misoprostol, diethylamine salicylate, etodolac, etoricoxib, aceclofenac, aspirin, benzydamine hydrochloride, celecoxib, coal tar with salicylic acid and sulphur, compound preparations with salicylic acid, diclofenac, diclofenac with misoprostol, diethylamine salicylate, etodolac, etoricoxib, felbinac, ibuprofen, ketoprofen, ketorolac trometamol, meloxicam, nabumetone, naproxen, nefopam hydrochloride, piroxicam, salicylic acid, sulindac  **Paracetamol:** paracetamol, paracetamol with metoclopramide  **Other pain drugs:** amitriptyline, capsaicin, carbamazepine, duloxetine, gabapentin, ketamine, lidocaine, menthol, nortriptyline, pregabalin  **Reflux medication:** esomeprazole, lansoprazole, nizatidine, omeprazole, pantoprazole, ranitidine, sucralfate  **Other palliative care drugs:** artificial saliva, ascorbic acid, baclofen, benzocaine, cannabinoids, carbocisteine, citric acid, compound alginic acid preparations, compound, dicycloverine hydrochloride, fluocinolone acetonide, levetiracetam, mebeverine hydrochloride, methylprednisolone, phenobarbital, primidone, salicylic acid and mucopolysaccharide polysulfate, sotalol hydrochloride, tizanidine hydrochloride, zopiclone  **Lipid-Lowering Drugs:** atorvastatin, bezafibrate, colestipol hydrochloride, colestyramine, ezetimibe, rosuvastatin, simvastatin  **Steroids:** beclometasone dipropionate, beclometasone dipropionate and formoterol fumarate, betamethasone, budesonide, clobetasol propionate, clobetasone butyrate, combined rectal preparations with prednisolone, compound rectal preparations with hydrocortisone, desmopressin, dexamethasone, fludrocortisone acetate, fludroxycortide, hydrocortisone, prednisolone  **Strong opioid:** alfentanil, buprenorphine, co-dydramol, diamorphine hydrochloride, dihydrocodeine tartrate, fentanyl, hydromorphone hydrochloride, meptazinol, methadone hydrochloride, morphine, morphine with cyclizine, oxycodone, oxycodone and naloxone, paracetamol with tramadol hydrochloride, tramadol hydrochloride  **Weak opioid:** co-codamol, co-codamol with buclizine hydrochloride, co-proxamol, codeine phosphate, pholcodine  **Other non-palliative care drugs: Any other prescribed medication not included in the defined drug categories.**  Missing Drug Name: Any prescription for which there was no entered drug name. |
| --- |

**Table S1: Multivariate analysis: Logistic regression of demographic, cancer type and temporal factors associated with likelihood of being prescribed a particular class of drug, vs. not being prescribed that class of drug, in the last year of life^[[1]](#footnote-1)^**

|  | Paracetamol  AOR^[[2]](#footnote-2)^ (95%CI) | NSAIDs  AOR (95%CI) | Any Opioid  AOR (95%CI) |
| --- | --- | --- | --- |
| Sex  Female (ref)  Male | **1.0**  **0.89 (0.86 to 0.93)** | **1.0**  **1.16 (1.10 to 1.23)** | **1.0**  **0.88 (0.86 to 0.91)** |
| Age  <65 (ref)  65-74  75-84  ≥85 | 1.0  0.97 (0.92 to 1.03)  1.02 (0.97 to 1.08)  **1.09 (1.02 to 1.16)** | 1.0  **1.33 (1.23 to 1.44)**  **1.26 (1.17 to 1.37)**  **1.42 (1.30 to 1.55)** | 1.0  **0.73 (0.70 to 0.76)**  **0.51 (0 .49 to 0.53)**  **0.37 (0.35 to 0.39)** |
| Cancer Type  Lung (ref)  Upper GI  Bowel  Breast & Ovarian  Prostate  Haematological  Other | 1.0  **0.92 (0.87 to 0.97)**  **0.93 (0.87 to 0.99)**  0.98 (0.91 to 1.06)  1.09 (0.99 to 1.20)  0.95 (0.88 to 1.02)  1.00 (0.94 to 1.06) | 1.0  **0.69 (0.64 to 0.74)**  **0.71 (0.65 to 0.78)**  0.92 (0 .83 to 1.02)  **0.63 (0 .54 to 0.73)**  **0.82 (0.75 to 0.91)**  **0.90 (0.83 to 0.97)** | 1.0  **0.86 (0.82 to 0.90)**  **0.85 (0.80 to 0.89)**  **0.75 (0.71 to 0.80)**  **1.12 (1.04 to 1.20)**  **0.75 (0.71 to 0.80)**  **0.84 (0.80 to 0.88)** |
| Rurality Grouped**^[[3]](#footnote-3)^**  Urban(ref)  Accessible  Remote | 1.0  0.99 (0.94 to 1.04)  1.02 (0.95 to 1.10) | 1.0  0.97 (0.90 to 1.04)  0.91 (0.83 to 1.00) | **1.0**  **1.04 (1.00 to 1.08)**  1.02 (0.96 to 1.08) |
| Deprivation**^[[4]](#footnote-4)^**  SIMD5 1 (ref)  SIMD5 2  SIMD5 3  SIMD5 4  SIMD5 5 | 1.0  1.01 (0.94 to 1.07)  0.98 (0.92 to 1.05)  0.94 (0 .89 to 1.00)  0.87 (0.81 to 0.93) | 1.0  0.94 (0.87 to 1.02)  0.96 (0.88 to 1.04)  0.91 (0.84 to 0.99)  0.87 (0.80 to 0.95) | 1.0  0.94 (0.90 to 0.99)  0.87 (0.83 to 0.92)  0.96 (0.92 to 1.01)  0.95 (0.90 to 1.00) |
| Time between diagnosis and death  0-12 weeks before death (ref)  13-25 weeks before death  26-38 weeks before death  39-51 weeks before death  ≥52 weeks before death | 1.0  1.04 (0.98 to 1.11)  **1.07 (1.00 to 1.15)**  **1.16 (1.08 to 1.24)**  **1.08 (1.03 to 1.14)** | 1.0  0.97 (0.90 to 1.05)  **0.77 (0.70 to 0.84)**  **0.67 (0.61 to 0.74)**  **0.66 (0.62 to 0.71)** | 1.0  **1.48 (1.41 to 1.56)**  **1.69 (1.60 to 1.78)**  **1.75 (1.66 to 1.85)**  **1.80 (1.73 to 1.88)** |

|  | Weak Opioid  OR (95%CI) | Strong Opioid  OR (95%CI) | Breakthrough medication  OR (95%CI) | Adjuvant analgesia  OR (95%CI) |
| --- | --- | --- | --- | --- |
| Sex  Female (ref)  Male | **1.0**  **0.91 (0.85 to 0.97)** | **1.0**  **0.89 (0.86 to 0.92)** | **1.0**  **1.54 (1.40 to 1.69)** | 1.0  **0.85 (0.80 to 0.91)** |
| Age  <65 (ref)  65-74  75-84  ≥85 | 1.0  0.98 (0.90 to 1.08)  1.01 (0.92 to 1.10)  **0.88 (0.80 to 0.98**) | 1.0  **0.71 (0.68 to 0.74)**  **0.44 (0.42 to 0.46)**  **0.29 (0.28 to 0.31)** | 1.0  **1.40 (1.22 to 1.60)**  **1.57 (1.39 to 1.79)**  **1.45 (1.25 to 1.68)** | 1.0  **0.75 (0.69 to 0.81)**  **0.55 (0.51 to 0.60)**  **0.37 (0.33 to 0.41)** |
| Cancer Type  Lung (ref)  Upper GI  Bowel  Breast & Ovarian  Prostate  Haematological  Other | 1.0  **0.80 (0.73 to 0.87)**  0.97 (0.87 to 1.07)  0.89 (0.79 to 1.00)  **1.24 (1.07 to 1.45**)  0.91 (0.81 to 1.03)  **0.86 (0.78 to 0.94)** | 1.0  **0.89 (0.85 to 0.93)**  **0.82 (0.78 to 0.87)**  **0.73 (0.68 to 0.78)**  **1.09 (1.01 to 1.18)**  **0.71 (0.66 to 0.77)**  **0.84 (0.80 to 0.89)** | 1.0  **1.30 (1.15 to 1.47)**  **1.18 (1.02 to 1.36)**  1.09 (0.91 to 1.31)  0.81 (0.65 to 1.01)  **1.60 (1.39 to 1.86)**  **1.18 (1.03 to 1.34)** | 1.0  **0.69 (0.62 to 0.76)**  **0.67 (0.60 to 0.75)**  **0.72 (0.64 to 0.81)**  1.03 (0.89 to 1.19)  **1.13 (1.01 to 1.26)**  1.01 (0.93 to 1.11) |
| Rurality Grouped**^[[5]](#footnote-5)^**  Urban(ref)  Accessible  Remote | 1.0  0.92 (0.85 to 1.00)  1.08 (0.96 to 1.20) | 1.0  **1.07 (1.02 to 1.11)**  1.00 (0.94 to 1.07) | 1.0  1.05 (0.94 to 1.18)  **0.76 (0.64 to 0.91**) | 1.0  1.03 (0.95 to 1.12)  0.92 (0.82 to 1.04) |
| Deprivation**^[[6]](#footnote-6)^**  SIMD5 1 (ref)  SIMD5 2  SIMD5 3  SIMD5 4  SIMD5 5 | 1.0  **0.79 (0.72 to 0.88)**  0.90 (0.82 to 1.00)  **0.83 (0.75 to 0.91)**  **0.83 (0.74 to 0.92)** | 1.0  0.99 (0.94 to 1.04)  **0.86 (0.82 to 0.91)**  1.01 (0.96 to 1.06)  0.98 (0.93 to 1.04) | 1.0  0.88 (0.78 to 1.00)  **0.68 (0.59 to 0.78)**  **0.74 (0.65 to 0.84)**  **0.61 (0.53 to 0.70)** | 1.0  0.91 (0.83 to 1.01)  1.03 (0.94 to 1.14)  **0.82 (0.74 to 0.90)**  **0.88 (0.79 to 0 .98)** |
| Time between diagnosis and death  0-12 weeks before death (ref)  13-25 weeks before death  26-38 weeks before death  39-51 weeks before death  ≥52 weeks before death | 1.0  0.92 (0.83 to 1.01)  0.91 (0.82 to 1.01)  **0.78 (0.70 to 0.87)**  **0.72 (0.66 to 0.78)** | **1.0**  **1.81 (1.71 to 1.93)**  **2.13 (2.01 to 2.27)**  **2.31 (2.17 to 2.46)**  **2.44 (2.32 to 2.56)** | **1.0**  **1.28 (1.13 to 1.46)**  1.05 (0.91 to 1.22**)**  **1.16 (1.00 to 1.35)**  **1.28 (1.15 to 1.42)** | 1.0  **1.13 (1.01 to 1.26)**  0.95 (0.85 to 1.07)  **1.18 (1.05 to 1.32)**  **1.61 (1.48 to 1.75)** |

|  | Laxatives  OR (95%CI) | Anti-Emetics  OR (95%CI) | Anxiolytics  OR (95%CI) | Anti-muscarinic  OR (95%CI) |
| --- | --- | --- | --- | --- |
| Sex  Female (ref)  Male | **1.0**  **1.11 (1.05 to 1.18)** | **1.0**  **0.70 (0.66 to 0.74)** | **1.0**  **0.75 (0.70 to 0.81)** | 1.0  1.03 (0.93 to 1.14) |
| Age  <65 (ref)  65-74  75-84  ≥85 | 1.0  1.01 (0.93 to 1.09)  0.99 (0.91 to 1.07)  0.97 (0.89 to 1.06) | **1.0**  **0.72 (0.67 to 0.78)**  **0.62 (0.57 to 0.67)**  **0.47 (0.43 to 0.52)** | **1.0**  **0.79 (0.73 to 0.86)**  **0.54 (0.50 to 0.59)**  **0.45 (0.41 to 0.51)** | **1.0**  **0.71 (0.63 to 0.80)**  **0.49 (0.43 to 0.55)**  **0.46 (0.40 to 0.54)** |
| Cancer Type  Lung (ref)  Upper GI  Bowel  Breast & Ovarian  Prostate  Haematological  Other | 1.0  **1.24 (1.15 to 1.35)**  1.04 (0.95 to 1.15)  **1.50 (1.35 to 1.66)**  **1.60 (1.42 to 1.82)**  1.08 (0.97 to 1.21)  **1.24 (1.14 to 1.35)** | 1.0  **1.83 (1.69 to 1.98)**  **1.52 (1.38 to 1.66)**  **0.97 (0.87 to 1.08)**  **1.04 (0.88 to 1.22)**  **0.83 (0.73 to 0.95)**  **0.96 (0.87 to 1.05)** | 1.0  **0.84 (0.77 to 0.92)**  **0.65 (0.58 to 0.73)**  **0.63 (0.56 to 0.71)**  **0.60 (0.50 to 0.74)**  **0.66 (0.58 to 0.75)**  **0.82 (0.75 to 0.91**) | 1.0  **1.32 (1.16 to 1.51)**  **1.45 (1.25 to 1.69)**  0.98 (0.81 to 1.20)  1.05 (0.81 to 1.36)  **0.79 (0.63 to 0.98)**  **1.18 (1.02 to 1.37)** |
| Rurality Grouped**^[[7]](#footnote-7)^**  Urban(ref)  Accessible  Remote | 1.0  **1.27 (1.19 to 1.36)**  1.09 (0.99 to 1.20**)** | 1.0  1.01 (0.94 to 1.09)  **1.11 (1.01 to 1.23)** | 1.0  **0.83 (0.76 to 0.91)**  **0.83 (0.74 to 0.95)** | **1.0**  **1.14 (1.01 to 1.28)**  **0.71 (0.58 to 0.86)** |
| Deprivation**^[[8]](#footnote-8)^**  SIMD5 1 (ref)  SIMD5 2  SIMD5 3  SIMD5 4  SIMD5 5 | **1.0**  **1.14 (1.04 to 1.25)**  0.99 (0.90 to 1.08)  **1.10 (1.00 to 1.20)**  1.09 (0.99 to 1.19) | **1.0**  **1.17 (1.06 to 1.28**)  1.02 (0.93 to 1.13)  **1.22 (1.12 to 1.34)**  **1.17 (1.06 to 1.30)** | **1.0**  **0.81 (0.73 to 0.90)**  **0.86 (0.78 to 0.96)**  **0.88 (0.80 to 0.98)**  **1.14 (1.03 to 1.26)** | 1.0  1.00 (0.85 to 1.17)  0.99 (0.84 to 1.16)  1.16 (0.99 to 1.35)  1.18 (1.00 to 1.38) |
| Time between diagnosis and death  0-12 weeks before death (ref)  13-25 weeks before death  26-38 weeks before death  39-51 weeks before death  ≥52 weeks before death | 1.0  **1.12 (1.03 to 1.22)**  **1.16 (1.06 to 1.27)**  **1.27 (1.16 to 1.40)**  1.06 (0.99 to 1.14) | **1.0**  **1.70 (1.54 to 1.86)**  **1.78 (1.61 to 1.96)**  **2.15 (1.95 to 2.37)**  **1.71 (1.58 to 1.85)** | **1.0**  **1.21 (1.09 to 1.34)**  **1.27 (1.14 to 1.41)**  0.99 (0.88 to 1.12)  1.07 (0.98 to 1.16) | 1.0  **1.40 (1.21 to 1.62**)  0.96 (0.81 to 1.14)  1.03 (0.87 to 1.22)  1.07 (0.94 to 1.21) |

|  | Antibiotics  OR (95%CI) | Antidepressants  OR (95%CI) | Other Palliative Care Drugs  OR (95%CI) | Other cancer drugs  OR (95%CI) |
| --- | --- | --- | --- | --- |
| Sex  Female (ref)  Male | **1.0**  **0.91 (0.86 to 0.96)** | **1.0**  **0.77 (0.72 to 0.83)** | 1.0  0.95 (0.89 to 1.02) | **1.0**  **2.72 (2.51 to 2.95)** |
| Age  <65 (ref)  65-74  75-84  ≥85 | 1.0  0.97 (0.90 to 1.04)  **0.895 (0.83 to 0.96)**  **0.90 (0.83 to 0.97)** | 1.0  0.95 (0.86 to 1.04)  **0.73 (0.66 to 0.80)**  **0.71 (0.64 to 0.79)** | 1.0  0.94 (0.86 to 1.02)  **0.73 (0.67 to 0.79)**  **0.64 (0.58 to 0.71)** | **1.0**  **1.31 (1.17 to 1.46)**  **1.94 (1.75 to 2.15)**  **2.38 (2.13 to 2.66)** |
| Cancer Type  Lung (ref)  Upper GI  Bowel  Breast & Ovarian  Prostate  Haematological  Other | 1.0  **0.74 (0.69 to 0.80)**  **0.77 (0.71 to 0.84)**  **0.90 (0.82 to 0.99)**  0.94 (0.83 to 1.07)  1.06 (0.97 to 1.16)  **0.90 (0.84 to 0.97)** | 1.0  1.02 (0.93 to 1.12)  1.01 (0.90 to 1.13)  **1.16 (1.03 to 1.31)**  **0.68 (0.54 to 0.84)**  **1.27 (1.12 to 1.43)**  **0.86 (0.78 to 0.96)** | 1.0  **1.10 (1.01 to 1.20)**  **0.73 (0.65 to 0.82**)  1.05 (0.93 to 1.18)  **0.56 (0.45 to 0.68)**  1.09 (0.97 to 1.23)  **1.13 (1.03 to 1.24)** | 1.0  **1.18 (1.06 to 1.30)**  1.10 (0.98 to 1.23)  **3.69 (3.26 to 4.18)**  **3.13 (2.80 to 3.50)**  **1.46 (1.30 to 1.65)**  **1.17 (1.05 to 1.30)** |
| Rurality Grouped**^[[9]](#footnote-9)^**  Urban(ref)  Accessible  Remote | 1.0  1.05 (0.98 to 1.12)  **1.33 (1.22 to 1.45)** | **1.0**  **0.65 (0.59 to 0.71)**  **0.80 (0.71 to 0.91)** | 1.0  0.99 (0.91 to 1.07)  1.05 (0.94 to 1.18) | **1.0**  **0.85 (0.78 to 0.92)**  0.91 (0.81 to 1.01) |
| Deprivation**^[[10]](#footnote-10)^**  SIMD5 1 (ref)  SIMD5 2  SIMD5 3  SIMD5 4  SIMD5 5 | 1.0  0.98 (0.90 to 1.06)  0.94 (0.87 to 1.02)  **0.91 (0.84 to 0.99)**  1.06 (0.98 to 1.16) | 1.0  0.79 (0.71 to 0.89)  0.95 (0.85 to 1.06)  1.02 (0.92 to 1.13)  1.16 (1.04 to 1.29) | 1.0  1.03 (0.93 to 1.14)  0.94 (0.85 to 1.04)  0.97 (0.87 to 1.07)  1.04 (0.94 to 1.16) | 1.0  1.11 (0.99 to 1.24)  **1.36 (1.22 to 1.51)**  **1.41 (1.27 to 1.55)**  **1.35 (1.22 to 1.50)** |
| Time between diagnosis and death  0-12 weeks before death (ref)  13-25 weeks before death  26-38 weeks before death  39-51 weeks before death  ≥52 weeks before death | 1.0  0.95 (0.88 to 1.03)  0.92 (0.84 to 1.0)  **0.84 (0.77 to 0.92)**  **0.91 (0.86 to 0.97)** | 1.0  **0.89 (0.81 to 0.99)**  **0.65 (0.57 to 0.73)**  **0.67 (0.59 to 0.76)**  **0.82 (0.75 to 0.89)** | 1.0  0.97 (0.88 to 1.08)  **1.24 (1.12 to 1.38)**  **1.13 (1.01 to 1.26)**  1.02 (0.94 to 1.11) | 1.0  **0.75 (0.67 to 0.84)**  1.01 (0.90 to 1.12)  1.06 (0.95 to 1.19)  1.06 (0.98 to 1.15) |

|  | Anti-hypertensive  OR (95%CI) | Statins and cholesterol-lowering drugs  OR (95%CI) | Bone protection  OR (95%CI) | Enteral Nutrition OR (95%CI) |
| --- | --- | --- | --- | --- |
| Sex  Female (ref)  Male | 1.0  1.01 (0.98 to 1.05) | **1.0**  **1.23 (1.17 to 1.31)** | **1.0**  **0.49 (0.45 to 0.54)** | **1.0**  **1.13 (1.06 to 1.21)** |
| Age  <65 (ref)  65-74  75-84  ≥85 | **1.0**  **2.14 (2.02 to 2.27)**  **2.63 (2.49 to 2.78)**  **2.87 (2.71 to 3.04)** | **1.0**  **2.01 (1.83 to 2.21)**  **2.02 (1.84 to 2.22)**  **1.83 (1.66 to 2.03)** | **1.0**  **1.76 (1.49 to 2.07)**  **2.51 (2.16 to 2.92)**  **4.11 (3.53 to 4.79)** | 1.0  0.92 (0.84 to 1.00)  0.92 (0.85 to 1.00**)**  **0.79 (0.71 to 0.87)** |
| Cancer Type  Lung (ref)  Upper GI  Bowel  Breast & Ovarian  Prostate  Haematological  Other | 1.0  1.04 (0.99 to 1.08)  **0.90 (0.86 to 0.95)**  1.00 (0.94 to 1.06)  **0.85(0.79 to 0.93)**  **1.20 (1.14 to 1.27)**  0.99 (0.94 to 1.04) | 1.0  **0.86 (0.79 to 0.92)**  0.97 (0.89 to 1.06)  **1.29 (1.17 to 1.43)**  **0.83 (0.72 to 0.97)**  1.05 (0.95 to 1.16)  **0.85 (0.78 to 0.92)** | 1.0  0.97 (0.86 to 1.10)  **0.81 (0.70 to 0.93)**  **2.02 (1.78 to 2.29)**  0.85 (0.65 to 1.11)  **1.44 (1.25 to 1.66)**  **1.15 (1.02 to 1.31)** | 1.0  **1.83 (1.69 to 1.99**)  **1.14 (1.03 to 1.27)**  **0.65 (0.57 to 0.75)**  0.90 (0.77 to 1.01)  0.88 (0.77 to 1.01)  1.03 (0.93 to 1.13) |
| Rurality Grouped**^[[11]](#footnote-11)^**  Urban(ref)  Accessible  Remote | **1.0**  **1.04 (1.00 to 1.08)**  0.96 (0.91 to 1.01) | 1.0  0.93 (0.87 to 1.00)  **0.79 (0.72 to 0.88)** | **1.0**  **0.86 (0.78 to 0.95)**  **0.57 (0.49 to 0.67)** | **1.0**  **1.16 (1.07 to 1.25)**  **1.48 (1.33 to 1.63)** |
| Deprivation**^[[12]](#footnote-12)^**  SIMD5 1 (ref)  SIMD5 2  SIMD5 3  SIMD5 4  SIMD5 5 | **1.0**  **1.07 (1.02 to 1.13)**  **1.07 (1.02 to 1.12)**  **1.10 (1.04 to 1.15)**  **1.06 (1.01 to 1.12)** | 1.0  1.03 (0.95 to 1.13)  1.06 (0.97 to 1.16)  0.99 (0.91 to 1.08)  1.08 (0.99 to 1.18) | **1.0**  **1.18 (1.03 to 1.36)**  **1.19 (1.04 to 1.36)**  **1.44 (1.27 to 1.63)**  0.98 (0.85 to 1.13) | 1.0  0.92 (0.83 to 1.02)  0.94 (0.84 to 1.05)  1.03 (0.93 to 1.14)  0.92 (0.83 to 1.01) |
| Time between diagnosis and death  0-12 weeks before death (ref)  13-25 weeks before death  26-38 weeks before death  39-51 weeks before death  ≥52 weeks before death | **1.0**  **0.72 (0.69 to 0.75)**  **0.69 (0.65 to 0.72)**  **0.58 (0.55 to 0.62)**  **0.59 (0.57 to 0.61)** | **1.0**  **0.71 (0.66 to 0.77)**  **0.63 (0.58 to 0.69)**  **0.59 (0.534 0.65)**  **0.44 (0.41 to 0.47)** | **1.0**  **0.78 (0.69 to 0.89)**  **0.80 (0.69 to 0.92)**  **0.79 (0.68 to 0.92)**  **0.79 (0.72 to 0.87)** | **1.0**  **2.33 (2.10 to 2.58)**  **2.43 (2.18 to 2.70)**  **2.61 (2.34 to 2.92)**  **2.12 (1.94 to 2.33)** |

|  | Reflux medication  OR (95%CI) | Anticoagulation  OR (95%CI) | Inhalers  OR (95%CI) | Steroids  OR (95%CI) |
| --- | --- | --- | --- | --- |
| Sex  Female (ref)  Male | 1.0  0.97 (0.93 to 1.02) | **1.0**  **1.17 (1.10 to 1.25)** | 1.0  1.00 (0.94 to 1.05) | 1.0  0.99 (.94 to 1.05) |
| Age  <65 (ref)  65-74  75-84  ≥85 | 1.0  1.02 (0.96 to 1.08)  0.97 (0.92 to 1.03)  1.02 (0.96 to 1.10) | 1.0  **1.14 (1.03 to 1.26)**  **1.64 (1.50 to 1.80)**  **1.49 (1.34 to 1.65**) | 1.0  **1.12 (1.04 to 1.21)**  **1.20 (1.12 to 1.30)**  **0.84 (0.77 to 0.92**) | 1.0  **0.82 (0.76 to 0.89)**  **0.76 (0.71 to 0.82**  **0.57 (0.52 to 0.63)** |
| Cancer Type  Lung (ref)  Upper GI  Bowel  Breast & Ovarian  Prostate  Haematological  Other | 1.0  **1.32 (1.24 to 1.39**)  0.94 (0.87 to 1.00)  **0.79 (0.72 to 0.86)**  **0.79 (0.70 to 0.88)**  0.97 (0.89 to 1.05)  0.94 (0.88 to 1.00) | 1.0  **0.88 (0.81 to 0.96)**  **0.78 (0.70 to 0.86)**  **1.14 (1.02 to 1.27)**  0.91 (0.78 to 1.05)  1.00 (0.90 to 1.11)  **0.76 (0.70 to 0.84**) | 1.0  **0.44 (0.41 to 0.47)**  **0.26 (0.24 to 0.29)**  **0.35 (0.31 to 0.39)**  **0.18 (0.14 to 0.22)**  **0.31 (0.27 to 0.35)**  **0.44 (0.40 to 0.47)** | 1.0  **0.81 (0.74 to 0.87)**  **0.69 (0.63 to 0.76)**  **0.72 (0.65 to 0.81)**  **0.87 (0.75 to 0.99)**  **0.73 (0.65 to 0.81)**  1.01 (0.93 to 1.09) |
| Rurality Grouped**^[[13]](#footnote-13)^**  Urban(ref)  Accessible  Remote | 1.0  0.98 (0.93 to 1.04)  1.03 (0.95 to 1.10) | 1.0  1.00 (0.93 to 1.08)  0.95 (0.85 to 1.06) | **1.0**  **0.90 (0.83 to 0.97)**  **1.39 (1.27 to 1.52)** | **1.0**  **1.20 (1.11 to 1.28)**  **1.30 (1.18 to 1.43)** |
| Deprivation**^[[14]](#footnote-14)^**  SIMD5 1 (ref)  SIMD5 2  SIMD5 3  SIMD5 4  SIMD5 5 | **1.0**  **1.08 (1.01 to 1.16)**  **1.10 (1.03 to 1.18)**  **1.16 (1.09 to 1.24)**  **1.13 (1.05 to 1.21)** | **1.0**  0.98 (0.89 to 1.08)  1.00 (0.91 to 1.10)  1.02 (0 .93 to 1.12)  **1.12 (1.02 to 1.24)** | 1.0  **0.65 (0.60 to 0.70)**  **0.61 (0.57 to 0.67)**  **0.59 (0.55 to 0.64)**  **0.38 (0.35 to 0.42)** | 1.0  1.06 (0.96 to 1.16)  1.10 (1.00 to 1.21)  1.07 (0.98 to 1.17)  **1.24 (1.13 to 1.36)** |
| Time between diagnosis and death  0-12 weeks before death (ref)  13-25 weeks before death  26-38 weeks before death  39-51 weeks before death  ≥52 weeks before death | 1.0  0.99 (0.93 to 1.06)  1.04 (0.97 to 1.11)  0.99 (0.91 to 1.06)  1.05 (1.00 to 1.11) | 1.0  0.72 (0.65 to 0.79)  0.79 (0.72 to 0.88)  0.69 (0.61 to 0.77)  0.73 (0.68 to 0.80) | 1.0  **1.19 (1.11 to 1.29)**  **0.90 (0.83 to 0.99)**  **0.63 (0.57 to 0.70)**  **0.91 (0.85 to 0.97)** | 1.0  **1.26 (1.15 to 1.38)**  **1.27 (1.15 to 1.39)**  **1.26 (1.15 to 1.39)**  **1.26 (1.17 to 1.35)** |

1. Statistically significant results are indicated in **bold** font [↑](#footnote-ref-1)
2. AOR (95%CI): Adjusted Odds Ratio with 95% Confidence Interval [↑](#footnote-ref-2)
3. Assessed using Scottish Government Urban Rural (SEUR) Classification data ‘Urban’ comprises SEUR1&2, ‘Accessible’ comprises SEUR3&5 and ‘Remote’ comprises SEUR 4 & 6. 33 people had missing information for rurality and deprivation and have been excluded from this analysis. [↑](#footnote-ref-3)
4. Scottish Index of Multiple Deprivation (SIMD). Category 1 is most deprived, and category 5 is least deprived. 33 people had missing information for rurality and deprivation and have been excluded from this analysis. [↑](#footnote-ref-4)
5. Assessed using Scottish Government Urban Rural (SEUR) Classification data ‘Urban’ comprises SEUR1&2, ‘Accessible’ comprises SEUR3&5 and ‘Remote’ comprises SEUR 4 & 6. 33 people had missing information for rurality and deprivation and have been excluded from this analysis. [↑](#footnote-ref-5)
6. Scottish Index of Multiple Deprivation (SIMD). Category 1 is most deprived, and category 5 is least deprived. 33 people had missing information for rurality and deprivation and have been excluded from this analysis. [↑](#footnote-ref-6)
7. Assessed using Scottish Government Urban Rural (SEUR) Classification data ‘Urban’ comprises SEUR1&2, ‘Accessible’ comprises SEUR3&5 and ‘Remote’ comprises SEUR 4 & 6. 33 people had missing information for rurality and deprivation and have been excluded from this analysis. [↑](#footnote-ref-7)
8. Scottish Index of Multiple Deprivation (SIMD). Category 1 is most deprived, and category 5 is least deprived. 33 people had missing information for rurality and deprivation and have been excluded from this analysis. [↑](#footnote-ref-8)
9. Assessed using Scottish Government Urban Rural (SEUR) Classification data ‘Urban’ comprises SEUR1&2, ‘Accessible’ comprises SEUR3&5 and ‘Remote’ comprises SEUR 4 & 6. 33 people had missing information for rurality and deprivation and have been excluded from this analysis. [↑](#footnote-ref-9)
10. Scottish Index of Multiple Deprivation (SIMD). Category 1 is most deprived, and category 5 is least deprived. 33 people had missing information for rurality and deprivation and have been excluded from this analysis. [↑](#footnote-ref-10)
11. Assessed using Scottish Government Urban Rural (SEUR) Classification data ‘Urban’ comprises SEUR1&2, ‘Accessible’ comprises SEUR3&5 and ‘Remote’ comprises SEUR 4 & 6. 33 people had missing information for rurality and deprivation and have been excluded from this analysis. [↑](#footnote-ref-11)
12. Scottish Index of Multiple Deprivation (SIMD). Category 1 is most deprived, and category 5 is least deprived. 33 people had missing information for rurality and deprivation and have been excluded from this analysis. [↑](#footnote-ref-12)
13. Assessed using Scottish Government Urban Rural (SEUR) Classification data ‘Urban’ comprises SEUR1&2, ‘Accessible’ comprises SEUR3&5 and ‘Remote’ comprises SEUR 4 & 6. 33 people had missing information for rurality and deprivation and have been excluded from this analysis. [↑](#footnote-ref-13)
14. Scottish Index of Multiple Deprivation (SIMD). Category 1 is most deprived, and category 5 is least deprived. 33 people had missing information for rurality and deprivation and have been excluded from this analysis. [↑](#footnote-ref-14)
