# Supplementary material for: Community prescribing trends and prevalence in the last year of life, for people who die from cancer
Source: BMC Palliat Care. 2022 Jul 8;21:120. doi: 10.1186/s12904-022-00996-3 (PMC9264643; doi:10.1186/s12904-022-00996-3)
Supplement: Supplementary file 2 — Additional file 2. [file 12904_2022_996_MOESM2_ESM.docx]

# Supplemental Material S1: Description of Study Population Characteristics:

### Age and Gender

Gender was balanced between sexes; 47.7% of the cohort were female and 52.3% were male (Figure 1). Patients’ age was calculated at their date of death. The cohort age range was 20-101 years with a mean age of 74.2, mode of 78, range of 81 and standard deviation of 11.57.

Figure 1 Demography: Age and Gender

####

###

### Rurality

The majority of the cohort (65.0%) lived in urban settings. There was however still a considerable number who lived in rural but accessible (24.0%) and remote rural (9.6%) locations. 33 people in the cohort had missing rurality information (Figure 2).

Figure 2 Demography: Rurality and Rurality Grouped

###

###

### Deprivation

The cohort was relatively evenly distributed in terms of deprivation, with the exception of SIMD4, which was relatively over-represented (n=731, 29.9%). Most people lived in relatively less deprived places (SIMD4&5: n=1152, 47.10%) (Figure 3).

Figure 3 Demography: Deprivation and Cancer Type

#### Cancer Type

The most common cancer type was lung cancer, accounting for nearly a third (27.5%) of cases. This was followed by Upper GI malignancies (21%) and bowel cancers (12.4%). Breast, Prostate and Haematological malignancies were the next most common. Cancers with a low frequency were grouped together as ‘other’ (Figure 4).

Figure 4 Demography: Cancer Type
